# Period poverty in Nigeria: a scoping review of its drivers and implications among women of reproductive age

**DOI:** 10.1186/s12889-026-28966-x

**Published:** 2026-08-08

**Authors:** Hilary I. Okagbue, Olanma Okezie-Okafor, Juliet Ihuoma, Eyitayo Lawal, Success David, Ifeyinwa Anosike, Munachiso Elekwa, Emmanuella Nzeribe, Stephen O. Asaolu, Sunday Nto, Victory Ekpin, Afeez Olajire, Sunday Atobatele, Sidney Sampson

**Affiliations:** 1 Sydani Institute for Research and Innovation, Sydani Group, Abuja, Nigeria; 2 ACE Group, Abuja, Nigeria; 3 Sydani Initiative for International Development, Sydani Group, Abuja, Nigeria

**Keywords:** Period poverty, Period insecurity, Menstrual poverty, Menstrual hygiene management, Nigeria, Socioecological model

## Abstract

**Background:**

Period poverty, broadly defined as the lack of access to menstrual products, safe water and sanitation, and menstrual health education, disproportionately burdens women and girls in low- and middle-income countries. In Nigeria, an estimated 37 million women and girls are affected, yet evidence on the drivers and implications of period poverty remains fragmented across settings and population groups. This review systematically maps the existing evidence to inform policy, programming, and future research.

**Methods:**

A scoping review was conducted following Joanna Briggs Institute methodology and reported according to the PRISMA-ScR guideline. PubMed, Scopus, Google Scholar, and grey literature sources were searched for studies published between 2016 and 2026. Evidence was synthesized narratively and interpreted using the Socioecological Model.

**Results:**

Sixteen studies were included from 240 retrieved records after screening and eligibility assessment. Most were cross-sectional studies conducted among adolescent schoolgirls, predominantly in Southwest Nigeria. Five thematic domains were identified: menstrual hygiene practices and material access; menstrual health knowledge and sociocultural beliefs; period poverty and socioeconomic inequalities; psychosocial, health, and educational consequences; and WASH and institutional barriers. Economic and sociocultural barriers emerged as the most consistently documented drivers across included studies, with high menstrual product costs, structural poverty, and entrenched menstrual stigma reported across the majority of the evidence base. WASH-related barriers, particularly inadequate sanitation infrastructure in public schools, were the next most consistently documented domain, closely followed by gaps in menstrual health education. Health outcomes included reproductive and urinary tract infections, and significant school absenteeism and reduced academic participation were documented across studies. An emerging association between financial dependence on male partners for menstrual materials and exposure to sexual coercion was also identified.

**Conclusions:**

The available evidence suggests that period poverty in Nigeria is a multidimensional public health and social equity challenge requiring coordinated responses across the health, education, WASH, gender, and social protection sectors. However, the evidence base remains dominated by cross-sectional studies, highlighting the need for stronger longitudinal and intervention research.

**Supplementary Information:**

The online version contains supplementary material available at https://doi.org/10.1186/s12889-026-28966-x.

## Background

Menstruation is a normal and universal biological process experienced by an estimated 1.8–1.9 billion women of reproductive age worldwide every month [1]. Yet for millions of women and girls, particularly those in low- and middle-income countries (LMICs), this routine physiological event is entangled with deprivation, stigma, and missed opportunity [2, 3]. Period poverty refers to the inability to access the resources, services, knowledge, and supportive environments required to manage menstruation safely, hygienically, and with dignity. It extends beyond the inability to afford menstrual products and includes inadequate access to water, sanitation and hygiene (WASH) facilities, limited menstrual health education, menstrual stigma and discrimination, restrictive sociocultural norms, and broader socioeconomic inequalities that influence menstrual experiences [4–7]. The United Nations has recognised period poverty as a barrier to realising the rights to health, education, and dignity, with at least 614 million women and girls currently living in conflict-affected areas where menstrual needs are consistently unmet [7]. In contrast, menstrual hygiene management (MHM) refers specifically to the practical management of menstruation. According to UNICEF and the World Health Organization, MHM involves using clean menstrual materials that can be changed in privacy as often as necessary, having access to soap and water for washing the body, appropriate facilities for disposal or cleaning of menstrual materials, and access to accurate menstrual health information. While effective MHM is an essential component of menstrual health, period poverty encompasses the wider structural and social conditions that constrain individuals’ ability to achieve adequate menstrual hygiene management. For the purposes of this review, safe menstrual products refer to menstrual materials that are hygienic, effective, acceptable, and appropriate for repeated use or disposal in accordance with established menstrual health guidance. These include commercially manufactured disposable sanitary pads, tampons, menstrual cups, and reusable menstrual pads that are properly cleaned, dried, and maintained. In contrast, improvised menstrual materials include old cloths, rags, tissue paper, newspapers, foam, leaves, or other absorbent materials used without appropriate hygienic practices, which may increase the risk of leakage, discomfort, and poor menstrual hygiene management. Recognizing these distinctions is important because access to safe menstrual products represents one component of addressing period poverty, alongside adequate WASH facilities, menstrual health education, and supportive social and policy environments.

Accordingly, this review conceptualizes menstrual hygiene management as one dimension of period poverty rather than treating the two concepts as interchangeable. The review therefore examines the broader determinants of period poverty, including economic, institutional, sociocultural, environmental, and policy-related factors that collectively shape menstrual health experiences among women and girls in Nigeria.

Sub-Saharan Africa (SSA) bears a disproportionate share of the global period poverty burden, shaped by intersecting structural vulnerabilities: high poverty rates, chronic underinvestment in WASH infrastructure, deeply rooted menstrual taboos, and weak institutional frameworks for menstrual health governance [3, 8]. A multilevel analysis drawing on Performance Monitoring for Action survey data from six SSA countries found that more than half of women of reproductive age in five of the six countries had an unmet need for menstrual hygiene management (MHM) [3].

The situation in Nigeria paints a representative picture. As Africa’s most populous nation, with a population exceeding 220 million, a median age of approximately 18 years, and more than 37 million women and girls estimated to be affected by period poverty, the country’s menstrual health crisis is of continental significance [9, 10]. The consequences extend beyond menstrual hygiene alone, affecting educational participation, psychosocial well-being, and gender equity. In many parts of the country, girls miss school during menstruation because sanitary products are unaffordable, school toilets lack privacy and water access, or menstruation is still treated as a source of shame and silence [10–13]. Studies have also reported the use of unsafe menstrual materials among adolescents in low-income communities, alongside marked inequalities in menstrual health experiences between urban and rural populations [10, 13].

Despite growing advocacy and increased public attention in recent years, menstrual health in Nigeria is still weakly institutionalized within national policy and financing frameworks. Although the Federal Government validated Nigeria’s first National Policy on Menstrual Health and Hygiene Management in 2025 [14–16], there are concerns around implementation, sustainable financing, and the ability of the policy to ensure equitable access to menstrual health services and support across different populations and settings. Existing studies have examined issues such as access to menstrual products, menstrual hygiene practices, menstrual health knowledge, and the role of poverty and stigma, but there is still limited synthesis of how these factors intersect and shape the experiences of women and girls across different contexts in Nigeria. Despite growing research, no previous review has comprehensively synthesized how these determinants interact across ecological levels to influence period poverty in Nigeria.

The primary objective of this review is to map the scope, nature, and distribution of published and grey literature evidence on the drivers of period poverty among females of reproductive age in Nigeria. The specific objectives are to: (a) Map evidence on the availability, affordability, and types of menstrual products used by Nigerian women and girls and identify barriers to accessing commercial menstrual products. (b) Identify and characterize evidence on the role of WASH infrastructure, including access to water, sanitation facilities, and disposal systems, as a driver of period poverty in Nigerian settings. (c) Examine evidence on the state of menstrual health education and information access in Nigeria, including sources of menstrual information, knowledge gaps, and the relationship between education and MHM practices. (d) Describe the socioeconomic and sociocultural barriers, including poverty, stigma, cultural taboos, and gender norms, that shape menstrual health experiences in Nigeria. (e) Map the distribution of evidence across Nigeria’s six geopolitical zones, settings (urban, rural, school, community, humanitarian), and population groups. (f) Identify gaps in the existing evidence base to inform future research, policy, and practice.

This review therefore brings together existing evidence on the drivers and implications of period poverty among women of reproductive age in Nigeria, while also mapping menstrual hygiene practices, knowledge, and access-related challenges across different settings and population groups. In doing so, it contributes to the growing discourse on menstrual health by providing a broader picture of the structural, socioeconomic, cultural, and infrastructural factors shaping menstrual experiences in Nigeria. Understanding how these factors interact requires a framework that moves beyond single-level explanations. This review applies the Socioecological Model (SEM) as an analytical lens, recognizing that menstrual health outcomes are shaped by the interplay of individual, interpersonal, community, institutional, and structural influences, and that addressing period poverty in Nigeria demands responses that engage all these levels simultaneously. Beyond identifying what is already known, the review also highlights where important evidence gaps remain. The findings from this review are expected to support future research, inform context-specific menstrual health interventions, and contribute to policy discussions aimed at improving the health, dignity, education, and social participation of Nigerian women and girls.

## Methods

### Study design

This study adopts a scoping review design to map out the existing evidence on the drivers responsible for period poverty in Nigeria and its implications for the well-being of women of reproductive age within the Nigerian context. A scoping review is considered appropriate due to the broad and exploratory nature of the research topic and the need to identify key concepts, types of evidence and knowledge gaps [17]. This review was carried out based on the methodological guidance provided by the Joanna Briggs Institute (JBI) for scoping reviews. Thus, ensuring a systematic, transparent and reproducible process in identifying and synthesizing evidence across several study designs. The review was also reported in line with the PRISMA-ScR checklist, which ensured methodological rigor and transparency [18]. The use of this guideline supports detailed reporting of the search process, study selection and finding synthesis.

### Review framework

This review was conducted in accordance with the JBI methodological framework for scoping reviews [19]. The review questions were formulated using the Population-Concept-Context (PCC) framework. The population comprised females of reproductive age (10 to 49 years) residing in Nigeria across all settings, including schools, tertiary institutions, formal and informal economic sectors, and humanitarian settings. The concept encompassed period poverty and menstrual hygiene management (MHM) across four interconnected dimensions: access to menstrual products; access to WASH infrastructure; access to menstrual health education and information; and socioeconomic and sociocultural barriers to menstrual health. The context was geographically bounded to Nigeria across all six geopolitical zones, encompassing urban, rural, formal and humanitarian settings.

The SEM was applied both as an analytical mapping framework and as an interpretive framework to examine how determinants operating at different ecological levels interact to influence menstrual health experiences. Rather than considering these determinants independently, the review explored how structural conditions shape institutional environments, how institutional environments influence community norms and interpersonal relationships, and how these collectively affect individual menstrual experiences. Originally developed by Bronfenbrenner [20] and adapted for health promotion by McLeroy et al. [21], the SEM recognizes health outcomes are shaped by the interplay of influences across five levels: individual, interpersonal, community, institutional, and the policy and structural (Fig. 1). The SEM is applied here as an analytical mapping tool rather than an intervention framework, consistent with the position that it is most usefully employed to understand the interconnected determinants of health behaviour across multiple levels [22].

The PCC framework determined the boundaries of includable evidence while the adapted SEM structured its synthesis, together enabling this review to move beyond descriptive cataloguing toward a theoretically grounded account of how period poverty is produced and sustained across multiple levels of Nigerian society.

**Fig. 1:**
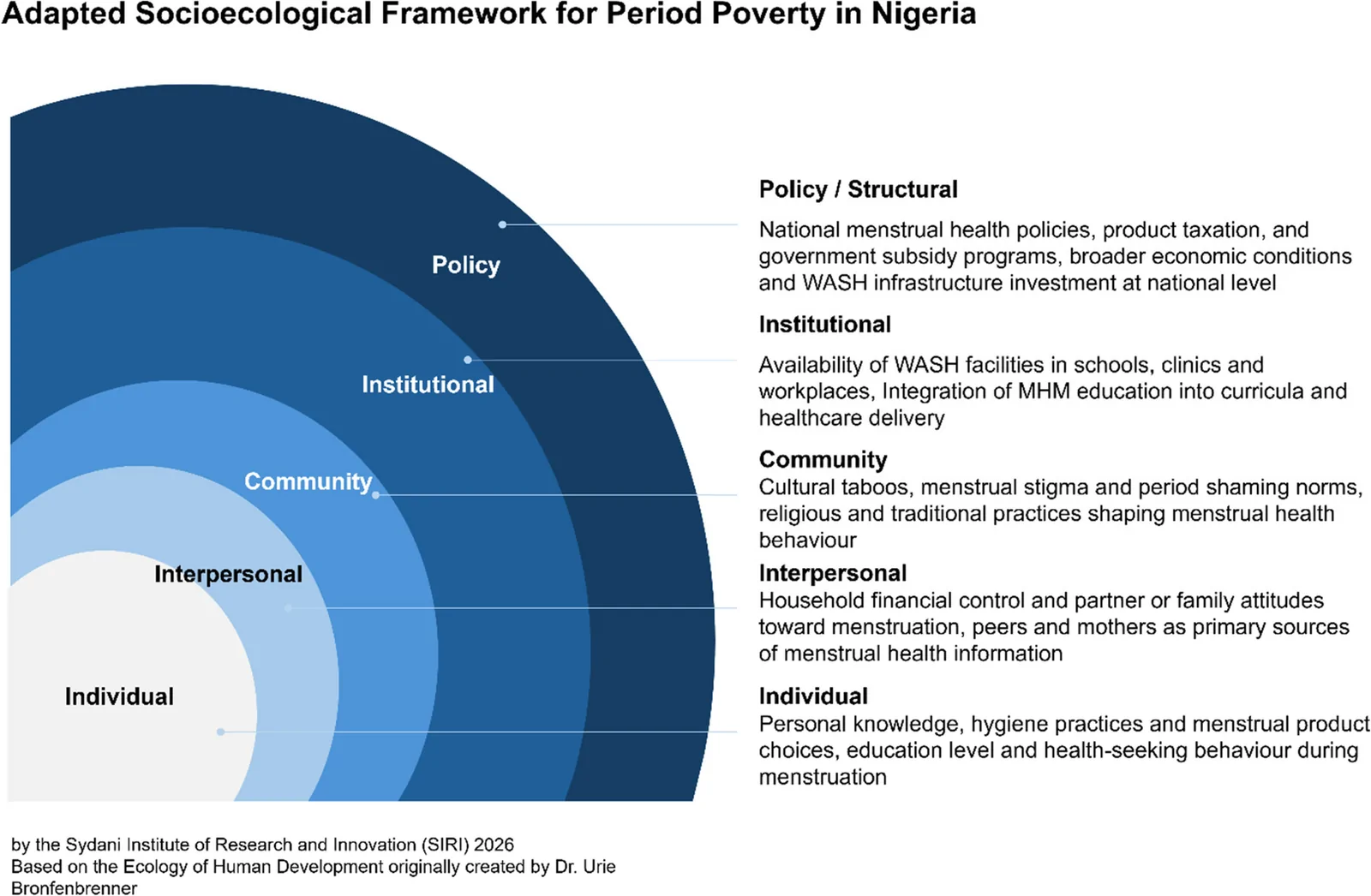
Adapted Socioecological Model illustrating the interacting individual, interpersonal, community, institutional, and structural determinants of period poverty among women of reproductive age in Nigeria. Adapted from Bronfenbrenner (1979)

### Eligibility criteria

Eligible studies were conducted in Nigeria and published in English between 2016 and 2026 in peer-reviewed journals and grey literature. The studies must involve females aged 10–49 years and address period poverty, menstrual hygiene, or menstrual hygiene management in Nigeria, including access to menstrual products, WASH facilities, menstrual health education and drivers, barriers and implications of period poverty. Review papers, pre-prints, studies conducted outside Nigeria, published in other languages and before 2016, studies in languages other than English, and studies not addressing the review objectives were excluded.

The review was restricted to studies in the stated study year because this period reflects the rapid expansion of menstrual health research following the adoption of the Sustainable Development Goals in 2015 and increasing international recognition of menstrual health as a public health, gender equity, and human rights issue. The review also sought to capture evidence that reflects contemporary menstrual health policies, educational practices, and socioeconomic conditions in Nigeria, including developments preceding the validation of the National Policy on Menstrual Health and Hygiene Management in 2025. Earlier studies were excluded to ensure that the synthesis reflected current evidence relevant to contemporary policy and programme development.

### Data source and search strategy

A comprehensive search strategy was developed in consultation with the Joanna Briggs Institute guidance for scoping reviews [19]. Electronic searches were conducted in PubMed, Scopus, and Google Scholar between 14 and 18 April 2026. Searches were limited to studies published in English. No restrictions were placed on study design. Reference lists of included studies and relevant review articles were also screened to identify additional eligible records.

Database-specific search strategies were developed by adapting the same core concepts to the indexing structure of each database. PubMed searches incorporated Medical Subject Headings (MeSH), whereas Scopus and Google Scholar used keyword combinations and Boolean operators. The complete reproducible search syntax for each database is presented in Supplementary Material 1.

PubMed was selected for its extensive indexing of public health and reproductive health literature and its support for Medical Subject Headings (MeSH)-enhanced searches. Scopus was selected for its breadth of coverage across public health, social sciences, and nursing. Google Scholar was used as a supplementary search source rather than a primary bibliographic database because it indexes a broad range of grey literature, including government reports, theses, dissertations, conference papers, institutional publications, and NGO reports that are often not indexed in traditional databases. Owing to the limitations of Google Scholar in supporting fully reproducible searches, screening was restricted to the first 200 results (20 pages of 10 records each) ranked by relevance, consistent with recommendations for systematic evidence synthesis. Screening was discontinued after 20 consecutive pages yielded no additional potentially eligible records, indicating diminishing retrieval of relevant evidence.

Grey literature was deliberately included to capture relevant non-peer-reviewed evidence and to ensure comprehensive coverage of period poverty across all four dimensions of the review framework. Grey literature searches were undertaken to minimise publication bias and capture relevant evidence not indexed in bibliographic databases. Sources included Google Scholar, official publications from the Federal Ministry of Health Nigeria, the Federal Ministry of Education, the National Bureau of Statistics, reports from international organizations such as UNICEF, UNFPA, and the World Health Organization, relevant NGO reports, institutional repositories of Nigerian universities, postgraduate theses and dissertations, policy documents, and conference proceedings where sufficient empirical data were reported. Google Scholar was additionally used to identify reports cited within eligible publications. Reference lists of all included studies and relevant policy documents were manually searched to identify additional eligible records.

Grey literature was screened using the same eligibility criteria as peer-reviewed studies. Only documents containing original empirical data relevant to period poverty among females aged 10–49 years in Nigeria were eligible for inclusion.

Search terms were combined using Boolean operators (AND, OR) to ensure systematic and comprehensive retrieval of relevant studies. The key terms included: “menstrual health,” “menstrual product access,” “menstrual education,” “menstrual hygiene,” “period poverty,” “menstrual poverty,” “period equity,” and “Nigeria.” For PubMed, MeSH terms were incorporated to enhance sensitivity, including “Menstruation,” “Hygiene,” and “Menstrual Hygiene Products.” Search strings were adapted for each database to reflect differences in indexing conventions and controlled vocabulary. The full search strings applied to each database are presented in Supplementary Material 1.

### Study selection

Consistent with the methodological approach adopted for this scoping review [17–19], four reviewers (OO, EL, SD, and JI) independently conducted blinded title, abstract, and full-text screening using the Rayyan systematic review platform to facilitate independent study selection and resolve screening disagreements [23]. Before formal screening commenced, the reviewers held a calibration exercise during which a random sample of records was independently screened and discussed to ensure a common understanding of the eligibility criteria and consistent interpretation of the inclusion and exclusion criteria. Any ambiguities identified during the calibration exercise were resolved through discussion before the full screening process commenced.

Screening was conducted in two sequential phases. During the first phase, titles and abstracts of all deduplicated records were independently screened and classified as “include,” “exclude,” or “maybe.” Records considered potentially eligible progressed to full-text assessment. During the second phase, full-text articles were independently assessed against the predefined eligibility criteria, with particular attention given to whether each study substantively addressed one or more dimensions of period poverty rather than merely mentioning menstruation.

Disagreements between reviewers at both screening stages were resolved through discussion. Where consensus could not initially be reached, the decision was resolved through majority agreement among the four reviewers. Reasons for exclusion during full-text screening were documented and are presented in the PRISMA flow diagram.

### Data extraction

A standardized data extraction form was developed in Microsoft Excel based on the review objectives and the Population–Concept–Context framework. Before formal data extraction commenced, the form was pilot-tested using a small sample of included studies to ensure consistency, completeness, and clarity of the extraction fields. Minor modifications were made following pilot testing to improve usability and ensure all relevant variables were captured. Two reviewers independently extracted data from all included studies, while three additional reviewers independently verified a random sample of extracted records for completeness and accuracy. Any discrepancies identified during verification were resolved through discussion until consensus was reached.

Evidence was additionally mapped across the five levels of the socioecological model, and health, psychosocial, and educational or economic outcomes reported by included studies were documented alongside author recommendations and noted study limitations.

### Data analysis and synthesis

Data analysis and synthesis followed a descriptive and thematic approach consistent with scoping review methodology [19]. Given the heterogeneity of study designs, populations, and outcome measures across included studies, quantitative pooling was not undertaken and findings were synthesised narratively, supported by summary tables.

Descriptive analysis was used to characterize included studies by design, geopolitical zone, setting, population group, and period poverty dimension addressed, mapping the volume, distribution, and thematic coverage of the existing evidence base. A thematic synthesis was subsequently applied to organise recurring patterns across the four review dimensions: menstrual product access, WASH and sanitation infrastructure, menstrual health education, and socioeconomic and sociocultural barriers.

Findings were further interpreted through the lens of the socioecological model across five levels of influence: individual, interpersonal, community, institutional, and policy and structural. This framing supported identification of how drivers of period poverty interact across levels and informed the development of multi-level policy and programme recommendations.

Consistent with scoping review methodology, no formal quality appraisal of included studies was undertaken, as the aim was to map the breadth and nature of available evidence rather than assess its strength.

## Results

For this scoping review, a total of 240 articles were retrieved from peer-reviewed databases, of which 32 duplicates were removed. Following deduplication, 208 articles were screened based on title and abstract. Full-text screening was conducted for 40 articles, resulting in the inclusion of 16 studies in the final review (see Fig. 2).

**Fig. 2:**
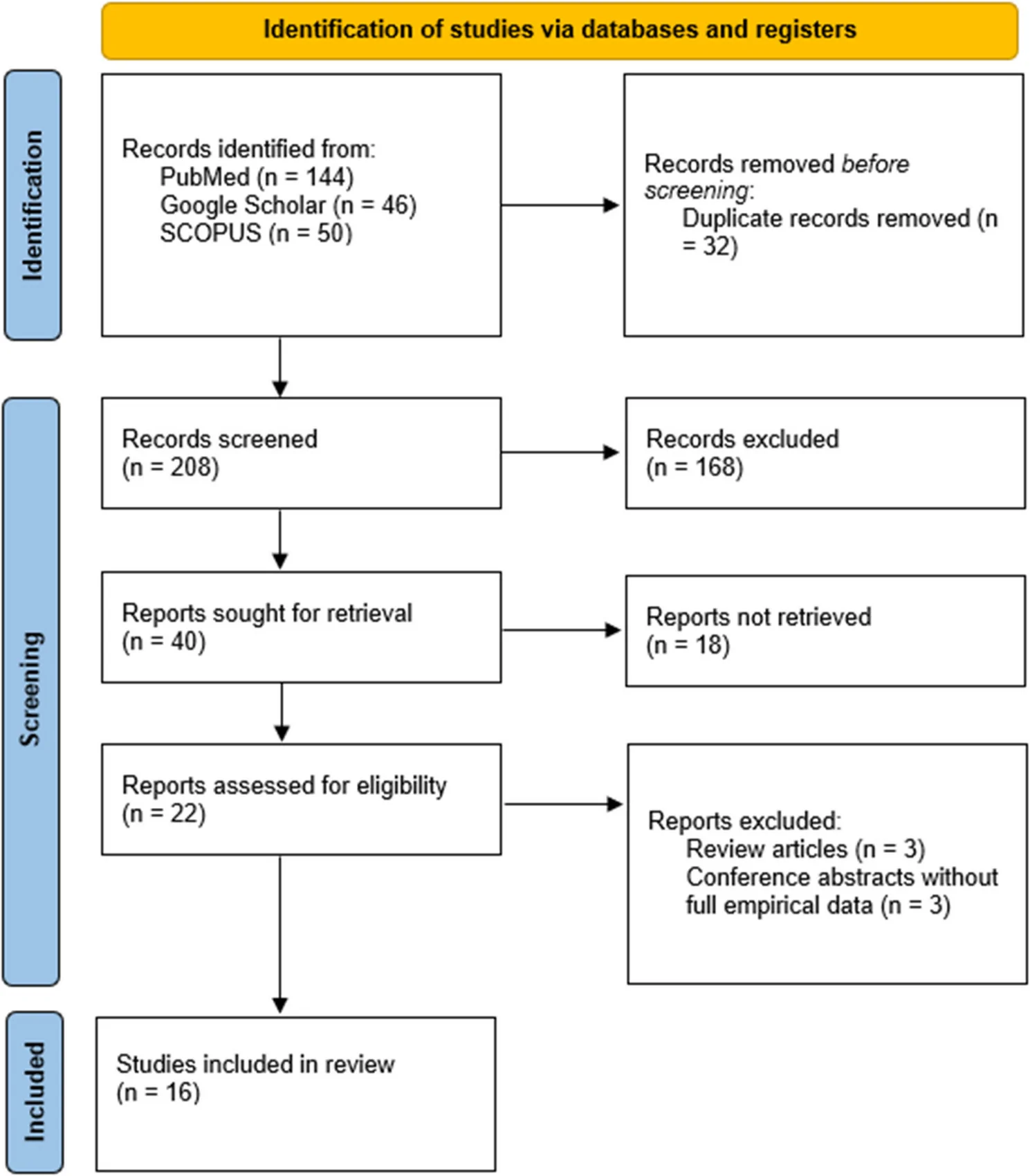
PRISMA-ScR flow diagram illustrating the identification, screening, eligibility assessment, and inclusion of studies in the scoping review, including reasons for exclusion at the full-text stage

All included studies were conducted across different regions of Nigeria, with the highest number in the Southwest (*n* = 6). Most of the studies focused on adolescent girls in school settings and explored menstrual hygiene practices, experiences and barriers to menstrual hygiene management. The key themes identified across the studies are menstrual hygiene practices and access to menstrual materials like sanitary pads, reusable cloths and improvised menstrual materials (*n* = 6); menstrual health knowledge, sociocultural beliefs, and stigma (*n* = 6); period poverty and socioeconomic inequalities (*n* = 5); menstruation-related educational, psychosocial, and health consequences (*n* = 5) and water, sanitation, and hygiene (WASH) and institutional support challenges (*n* = 5). Figure 3 thematically illustrates the themes in the review.

**Fig. 3:**
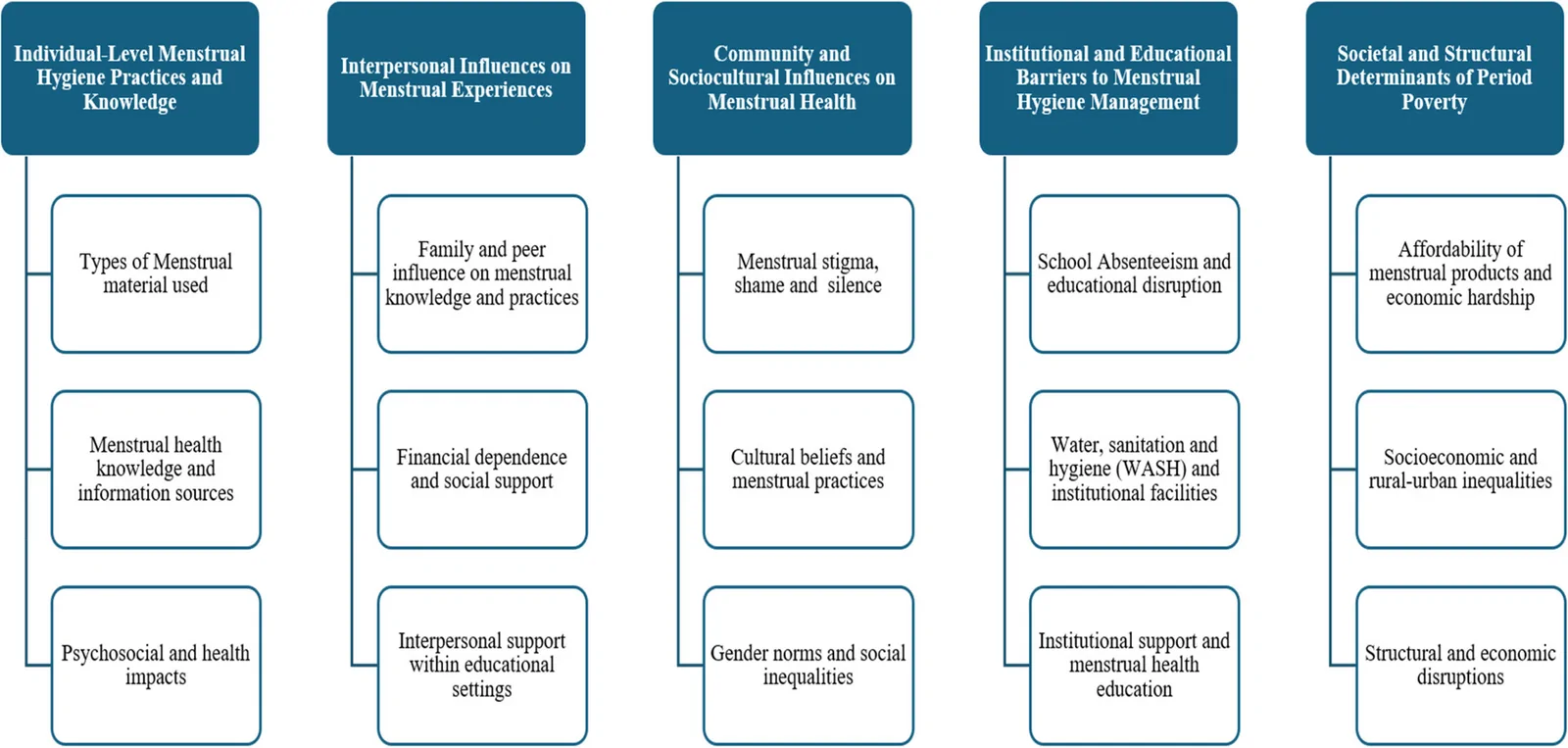
Summary of the five major thematic domains identified across the included studies and their alignment with the Socioecological Model used for evidence synthesis

### Theme 1: Individual-level menstrual hygiene practices and knowledge

Findings from the review reported varying individual-level factors affecting menstrual hygiene practices among girls and women in Nigeria. These factors included menstrual hygiene practices, menstrual health knowledge, types of menstrual materials used, and psychosocial and health experiences associated with menstruation [24–26].

#### Types of menstrual material used

Use of menstrual materials differs across included studies, with sanitary pad use ranging from 23.7% among adolescent girls to 95.0% among adolescent students. Higher sanitary pad usage was found among urban, private-school and tertiary-educated populations, while rural and lower-income groups depended more on reusable or improvised menstrual materials. In the study conducted among adolescent students in Ibadan, 95.0% of respondents reported using sanitary pads despite poor menstrual health knowledge among many participants [27]. Similarly, among female undergraduates in a private tertiary institution, 90.1% reported sanitary pad use, reflecting relatively better menstrual hygiene practices in tertiary educational settings [28]. However, several studies documented extensive reliance on alternative menstrual materials. In rural Katsina communities, only 36% of women used sanitary pads, while 44% relied on old cloths and rags for menstrual management. The study further reported widespread use of herbs and roots (63.5%) and dietary practices (27.3%) for menstrual management [24]. Likewise, the study conducted in rural Anambra communities found that only 23.7% of adolescent girls used disposable sanitary pads, while cloths/rags (40.6%) and tissue paper (32.3%) were more commonly used [29].

In Akure North, there was a nearly equal distribution between sanitary pad use (52%) and cloth or reusable pads (47.4%), suggesting continued dependence on reusable menstrual materials even where sanitary pads were available [25]. Similarly, the Akwa Ibom study reported that although sanitary pads were the most recognised (73.4%) and most used products (67.5%), more than half of the respondents (57.4%) also used reusable cloths [30]. The studies also saw significant rural–urban and institutional disparities in menstrual material use. In Cross River State, urban girls reported significantly higher sanitary pad use (91.7%) compared to rural girls (64.3%) (*p* = 0.001) [31]. Similarly, the Badagry study found that public school students were twice as likely to use non-pad alternatives compared to students in private schools (*p* < 0.001) [32]. These findings indicate persistent inequalities in access to safe menstrual materials across socioeconomic and geographical groups.

#### Menstrual health knowledge and information sources

Menstrual health knowledge was generally inadequate across studies despite widespread awareness of menstruation before menarche. Mothers, peers, and teachers were consistently identified as the primary sources of menstrual information. One of the included studies reported that most respondents had heard about menstruation before menarche, and many shown poor knowledge pertaining to appropriate menstrual hygiene practice [27]. Mothers, friends and teachers were identified as major sources of menstrual information. In addition, the review noted that sanitary pads were recognised and used, substantial gaps in menstrual hygiene knowledge persisted among respondents [30]. The review also identified peers and mothers as major sources of menstrual information.

The review identified higher levels of menstrual hygiene knowledge and safer menstrual practices among university students when compared to adolescents in a secondary school setting [28]. Therefore, suggesting that educational exposure influences menstrual health literacy and menstrual hygiene management practices. Furthermore, the review findings demonstrate improvement in menstrual health knowledge following menstrual hygiene education campaigns [33]. This suggests the importance of school-based menstrual health education interventions in improving menstrual literacy among adolescent girls. The review findings also stated that inadequate menstrual health knowledge was associated with poor menstrual hygiene practices, which further stresses the association between poor awareness and menstrual hygiene management outcomes [26].

#### Psychosocial and health impacts

Across the reviewed studies, menstruation was associated with a range of psychosocial and health challenges, including emotional distress, menstrual product insecurity, and concerns about poor menstrual hygiene. Studies in this review highlighted the psychosocial effects of menstruation among girls and women. Emotional stress and anxiety linked to the inability to afford menstrual products were identified as a psychosocial impact [34]. Some respondents in the study reported vulnerability to exploitation and coercive relationships associated with menstrual product insecurity. Economic hardship and limited menstrual support during the COVID-19 pandemic led to increased emotional distress and menstrual-related difficulties among girls and women [35]. The study highlighted the psychological burden associated with poor menstrual hygiene management during periods of economic crisis. The review also identified health-related concerns linked with poor menstrual hygiene practices. Identifying discomfort, irritation, and perceived susceptibility to infections among girls practising inadequate menstrual hygiene management [30]. These suggest that individual menstrual experiences are shaped not only by knowledge and practices but also by emotional and physical challenges associated with menstruation.

### Theme 2: Interpersonal influences on menstrual experiences

The studies reviewed identified several interpersonal factors that influence menstrual experiences and menstrual hygiene management among both girls and women in Nigeria. Family members, peers, teachers, and intimate partners played important roles in shaping menstrual knowledge, access to menstrual products, and psychosocial experiences during menstruation.

#### Family and peer influence on menstrual knowledge and practices

Family members and peers played a major role in shaping menstrual knowledge, attitudes, and experiences across the included studies. Mothers were identified as the main source of menstrual information and support for adolescent girls. Girls obtained menstrual education and guidance from their mothers, female relatives, friends and teachers [30]. These interpersonal relationships might have influenced menstrual knowledge, hygiene practices, and attitudes towards menstruation. The review also found that peer interactions shaped menstrual experiences among adolescent girls. Some girls reported fear of ridicule, teasing, and embarrassment from peers during menstruation, particularly within school environments [31]. Fear of staining school uniforms and negative peer reactions might have contributed to perceived reduced confidence, emotional discomfort, and withdrawal from social and academic participation during menstruation.

#### Financial dependence and social support

Findings for several studies showed that girls were dependent on their mothers or guardians for menstrual products, although some were dependent on their male partners for financial support when purchasing sanitary pads. This dependence primarily involved mothers and guardians, who were often the main providers of menstrual materials or financial support for purchasing sanitary pads [24, 29, 34].

Girls also relied on male partners for financial assistance to purchase menstrual products, highlighting how economic insecurity may expose girls to unequal power dynamics and potential vulnerability to coercion [34]. The high cost of sanitary pads was also identified as a major barrier to menstrual product usage in this review [24, 36]. Many women relied on old cloths and improvised menstrual materials because disposable sanitary pads were unaffordable. These illustrate that financial dependence for menstrual management is shaped by household poverty, broader socioeconomic inequality, and, in some cases, intimate partner dynamics.

#### Interpersonal support within educational settings

Teachers and school personnel also influenced menstrual experiences among adolescent girls. Teachers were identified as sources of menstrual information and support, inadequate menstrual support systems within schools, and limited effective assistance during menstruation [25]. Girls in schools with limited menstrual support reported greater challenges managing menstruation comfortably and privately. Hence, this demonstrates that interpersonal relationships significantly shape menstrual health experiences among girls and women in Nigeria by influencing menstrual knowledge, emotional support, access to menstrual materials, and psychosocial well-being.

### Theme 3: Community and sociocultural influences on menstrual health

The reviewed studies found that community beliefs, sociocultural norms, and menstrual stigma significantly shaped menstrual experiences among girls and women in Nigeria. Menstruation was frequently associated with secrecy, shame, silence, and restrictive cultural expectations, which may influence menstrual hygiene practices and communication surrounding menstruation.

#### Menstrual stigma, shame and silence

Several studies reported that menstruation remained highly stigmatised across many communities. Girls and women often avoided discussing menstruation openly due to fear of embarrassment, ridicule, and negative social judgment [37]. Menstruation was frequently associated with secrecy, embarrassment, and social restrictions. The review also reported the psychosocial impact associated with menstrual stigma [31]. Thereby noting that the fear of staining school uniforms, teasing from peers and embarrassment during menstruation might have contributed to school absenteeism and emotional discomfort. This indicates that menstrual stigma is embedded in social and cultural structures, which limits open discussions and access to menstrual support. Hence, this review illustrates that inadequate menstrual health knowledge and persistent sociocultural stigma continue to affect both menstrual hygiene management and menstrual experiences among both girls and women negatively in Nigeria.

#### Cultural beliefs and menstrual practices

The reviewed studies show the influence of cultural beliefs and traditional practices on menstrual experiences and hygiene behaviours. In rural Katsina communities, women reported the use of herbs, roots, and traditional remedies for menstrual management [24]. Some cultural restrictions and beliefs also shaped behaviours during menstruation, influencing hygiene practices and participation in social activities.

Findings from the review suggested that sociocultural expectations surrounding menstruation might have contributed to silence and limited open communication about menstrual health. Gender norms and cultural beliefs often restricted girls’ ability to seek menstrual support or discuss menstrual challenges comfortably within their communities [38].

#### Gender norms and social inequalities

The studies reviewed illustrated that menstrual experiences in Nigeria are shaped by socioeconomic and gender inequalities. Findings from one of the included studies highlighted the intersection between poverty, gender norms and menstrual health experiences among adolescent girls and women [38]. Thereby emphasising that menstrual health challenges are compounded by limited autonomy, unequal access to resources and sociocultural expectations around menstruation. The review found a significant difference in menstrual hygiene practices between students attending public and private schools [32]. Girls in public schools were more likely to use non-pad alternatives and experience inadequate menstrual hygiene management support when compared to students in private schools. The review also reported that girls from poorer backgrounds experience greater difficulty when managing menstruation in school due to limited access to menstrual products and inadequate support systems [31]. Overall, the review indicated that period poverty goes beyond the inability to buy sanitary products and is linked closely to broader socioeconomic inequalities, gender norms and institutional disparities which shape menstrual health experiences among both girls and women.

### Theme 4: Institutional and educational barriers to menstrual hygiene management

The reviewed studies identified institutional and educational barriers affecting menstrual hygiene management among girls and women in Nigeria. Inadequate water, sanitation, and hygiene (WASH) facilities, poor menstrual support systems within schools, and educational inequalities negatively influenced menstrual experiences and school participation.

#### School absenteeism and educational disruption

Menstruation-related school absenteeism was a recurring finding across studies and was commonly linked to menstrual pain, fear of staining, inadequate menstrual products, and poor school support systems. The findings from one of the studies in the review stated that many girls missed school due to fear of staining their uniform, teasing from peers, menstrual pain and inadequate menstrual support in schools [31]. Menstruation-related anxiety and embarrassment may reduce classroom participation and affected academic engagement. Inadequate access to sanitary pads and poor menstrual hygiene management facilities may contribute to school absenteeism and decrease classroom participation [11]. This indicates that girls without access to sanitary products were more likely to miss school during menstruation.

Educational disruption associated with menstruation was identified [32], as girls attending public schools reported poorer menstrual hygiene experiences and greater menstrual-related challenges when compared to students in private schools. This suggests that institutional disparities contribute to unequal educational experiences among adolescent girls.

#### Water, sanitation, hygiene and institutional facilities

Several studies reported inadequate sanitation infrastructure as a major challenge affecting menstrual hygiene management. Girls attending public schools reported poorer access to menstrual hygiene facilities compared to students in private schools [32]. The study highlighted inequalities in access to clean toilets, water supply, and private spaces for menstrual management, particularly among students from lower socioeconomic backgrounds. Similarly, the review reported that inadequate sanitation facilities and poor access to clean water are negatively associated with menstrual hygiene practices among rural adolescent girls [29]. Thereby illustrating difficulties in maintaining menstrual hygiene due to the limited availability of water and appropriate toilet facilities. Lack of privacy, fear of staining, and inadequate sanitation facilities discouraged girls from attending school during menstruation [31]. This illustrates the concerns regarding the absence of safe and private spaces for changing menstrual materials while in school.

#### Institutional support and menstrual health education

The included studies also highlighted limited institutional support for menstrual hygiene management across many schools and communities. The review found that there was inadequate menstrual hygiene support within schools despite relatively high awareness of menstrual hygiene practices [25]. Thus, identifying the need for improved school-based menstrual support systems and access to menstrual products. In addition, the review deduced that school-based menstrual health education interventions were associated with improved menstrual hygiene knowledge and awareness among adolescent girls [33]. Hence, emphasising the importance of integrating menstrual health education into school health programs so as to improve menstrual hygiene management practices.

### Theme 5: Societal and structural determinants of period poverty

The reviewed studies identified broader societal and structural factors contributing to period poverty and menstrual inequalities among girls and women in Nigeria. Economic hardship, socioeconomic inequalities, rural–urban disparities, and wider structural challenges significantly influenced access to menstrual products and menstrual hygiene management resources.

#### Affordability of menstrual products and economic hardship

Economic hardship emerged consistently across studies as a major driver of period poverty and limited access to menstrual products. The high cost of sanitary pads limited access to safe menstrual products among many girls and women, particularly within rural and low-income communities [24, 36]. In rural Katsina, the high cost of sanitary pads was identified as the leading barrier to pad use (39%), with married women disproportionately reporting cost-related challenges [24]. The study concluded that period poverty in the community was driven by economic hardship, sociocultural norms, and infrastructural limitations. A study among adolescent girls in Ibadan similarly found that economic insecurity limited access to menstrual products, with mothers serving as the primary source of menstrual materials or financial support for purchasing pads [34]. Some participants reported depending on male partners for menstrual product support, exposing them to risks of sexual coercion and exploitation.

Socioeconomic inequalities were also evident in the multicountry study on period poverty, which reported that 36.7% of women in Nigeria did not use sanitary pads and that poorer populations had significantly lower access to water and lockable menstrual hygiene management spaces [36]. Similarly, lack of money was identified as a major reason for the use of cloths, tissue paper, and other alternative menstrual materials in rural Anambra communities [29]. The study by Odey et al. reported that supply chain disruptions and rising product prices reduced access to menstrual materials, particularly among low-income girls and women [35]. Collectively, these findings highlight the strong influence of economic status on menstrual hygiene practices and access to menstrual products in Nigeria.

#### Socioeconomic and rural-urban inequalities

The reviewed studies showed significant socioeconomic inequalities in menstrual hygiene management practices and access to menstrual resources. Girls from lower socioeconomic backgrounds were less likely to use disposable sanitary pads and more likely to experience inadequate menstrual hygiene support [32]. The multi-country study on period poverty reported that over one-third of women in Nigeria did not use sanitary pads and that poorer populations had significantly lower access to water and lockable menstrual hygiene management spaces [36]. Rural–urban disparities were also evident, as urban girls consistently reported better access to sanitary pads and menstrual hygiene facilities compared to rural girls.

#### Structural and economic disruptions

The review further identified broader economic disruptions as factors associated with worsening menstrual product insecurity among vulnerable populations. During the COVID-19 pandemic, an increase in menstrual product prices, supply chain disruptions, and reduced accessibility to menstrual materials negatively affected girls and women from low-income households [35]. Economic crises were associated with worsening existing menstrual inequalities by increasing menstrual product insecurity and limiting access to menstrual hygiene resources. Collectively, these findings indicate that period poverty in Nigeria is closely linked to broader structural inequalities, economic hardship, and inadequate social support systems.

### Characteristics of the evidence base

Although no formal critical appraisal was conducted, the included evidence was predominantly derived from cross-sectional observational studies conducted among adolescent schoolgirls in educational settings. Most studies employed questionnaire-based surveys with convenience or school-based sampling approaches, and relatively few included rural populations or vulnerable groups such as women living with disabilities or internally displaced women. Consequently, the available evidence provides valuable descriptive information but remains limited in its capacity to establish causal relationships or evaluate intervention effectiveness. The review also identified a clear need for longitudinal cohort studies, implementation research, quasi-experimental evaluations, and randomized intervention studies capable of evaluating whether proposed menstrual health interventions improve educational, psychosocial, and health outcomes over time.

## Discussion

A key contribution of this review is demonstrating that the drivers of period poverty rarely operate independently. Rather, they interact across multiple levels of the Socioecological Model to reinforce menstrual disadvantage. For example, structural poverty limits household purchasing power, which in turn increases dependence on family members or intimate partners for menstrual products. This financial dependence may reinforce unequal gender power relations and increase vulnerability to exploitation. Simultaneously, inadequate investment in school WASH infrastructure restricts girls’ ability to manage menstruation safely, while prevailing community stigma discourages open discussion and help-seeking. These interacting influences collectively shape individual menstrual knowledge, product choices, educational participation, and psychosocial wellbeing. Consequently, interventions that target only one level such as providing menstrual products without improving school facilities, addressing stigma, or reducing structural poverty are unlikely to produce sustainable improvements. The findings therefore support the use of integrated, multisectoral interventions that simultaneously address structural, institutional, community, interpersonal, and individual determinants of menstrual health. However, interpretation of the review findings should consider the characteristics of the available evidence. The evidence base was dominated by descriptive cross-sectional studies, with comparatively few analytical or intervention studies. Consequently, the review primarily reflects patterns of association and reported experiences rather than causal relationships.

At the Individual level, menstrual practices and choices are tightly constrained by affordability and access. Our findings showed that women in rural regions rely on old clothes, rags, herbs and traditional methods rather than sanitary pads, with a clear association between the material used and reproductive health challenges. This is similar to a study in low-income communities, where half of the respondents could not afford pads and resorted to tissue paper, old towels, and newspapers, with nearly two-thirds reporting recent genitourinary infections, underlining the health risks of improvised materials [39]. Also, a study at the national level showed that around a third of women in Nigeria do not use sanitary pads, and poorer groups have markedly lower access to safe, lockable MHM spaces, confirming that wealth and infrastructure jointly shape menstrual options and dignity [36]. Although disposable sanitary pads were the predominant menstrual products reported across the included studies, environmentally sustainable menstrual products such as reusable menstrual pads and menstrual cups received relatively little attention. This limited emphasis likely reflects broader challenges related to affordability, availability, user awareness, cultural acceptability, and access to adequate water and sanitation facilities required for the safe use and maintenance of reusable products. Future menstrual health programmes and implementation research should evaluate the acceptability, cost-effectiveness, environmental sustainability, and long-term feasibility of different menstrual product options across diverse Nigerian settings. However, findings across the included studies were not uniform. Sanitary pad use ranged from 95% among adolescent students in Ibadan to as low as 23.7% in rural Anambra, a disparity that contrasts with reports from urban Nigerian settings and Ethiopia, where commercially produced pad use was considerably higher [29], suggesting that geographic location, school type, and socioeconomic background jointly determine menstrual material choice, and that national or regional averages risk masking significant sub-group inequalities. Critically, reusable clothes remained common even where pads were available, indicating that physical access alone is insufficient without sustained affordability. A cross-cutting finding across our studies was the persistent gap between menstrual awareness and actual knowledge of menstrual hygiene. We found that despite high pre-menarche awareness, adolescents showed poor knowledge of appropriate hygiene practices. This finding is consistent with a study among in-school girls in Abuja, which similarly found that despite the majority reporting good general knowledge of menstruation, significant practice gaps persisted [40], suggesting that knowledge-practice inconsistency is a structural problem rather than an isolated finding. This distinction matters because girls who are aware of menstruation but lack structured knowledge remain inadequately equipped to manage it safely. University students showed significantly better knowledge and safer practices than secondary school peers, confirming that progression through formal education was associated with better menstrual health literacy, and that interventions must be calibrated to educational level rather than treating awareness as sufficient. The psychosocial burden at this level was equally significant, with emotional distress, anxiety, and vulnerability to exploitation directly linked to menstrual product insecurity [24, 39], further compounded by COVID-19-related supply disruptions and rising product costs [35, 39].

At the interpersonal level, our findings show mothers were the primary source of menstrual information and material support, yet financial constraints frequently shifted this dependence toward male partners, exposing girls to unequal power dynamics and risks of sexual coercion [36]. This is a distinct and under-addressed implication of period poverty that extends beyond hygiene into gendered vulnerability and exploitation, and that warrants explicit attention in both policy and safeguarding frameworks. Within school settings, peer interactions reinforced stigma, with fear of teasing and uniform staining reducing academic confidence and participation, a pattern similar to other studies in which fear of staining and teasing drives girls’ self-isolation and absenteeism [2, 41]. However, a secondary analysis of menstrual stigma and mental health in LMICs found that while shame and stigma showed significant associations with mental health outcomes, the direct link between menstrual stigma and school absenteeism was not statistically proven in all settings, indicating that the relationship between stigma and absenteeism may be context-dependent and mediated by other factors such as infrastructure and economic conditions [42]. This divergent finding cautions against treating stigma as a universal and independent driver of absenteeism without accounting for local contextual factors. Teachers, though identified as information sources, operated within systems lacking adequate menstrual support structures, limiting their practical impact on girls’ menstrual experiences and underlining that interpersonal support cannot compensate for institutional deficiencies.

At the community level, sociocultural beliefs and stigma powerfully reinforce material deprivation. Narrative and psychosocial studies describe menstruation-related stigma as a form of structural discrimination, restricting open discussion, medical attention, and policy visibility while normalising secrecy, shame, and social exclusion [2, 43]. In the Nigerian context, another study emphasized that deep-seated menstrual taboos, combined with poor WASH facilities and lack of privacy, erode girls’ dignity in schools, workplaces, and religious spaces and weaken community support for menstrual health reforms [35]. Furthermore, both global research and Nigerian studies agree that period poverty is not just a private hygiene issue. Instead, it is a matter of gender equality, human rights, and social justice. This perspective highlights the need for public policies that address social stigma and economic inequality simultaneously, rather than treating them as separate problems [8, 44]. 

At the institutional level, inadequate WASH infrastructure was a central barrier, but the result points to an important distinction, that is, not only a rural infrastructure problem. Public school girls in urban and peri-urban settings consistently reported poorer access to clean toilets, water, and private changing spaces compared to private school counterparts, reflecting a systematic institutional inequality that disadvantages girls from lower socioeconomic backgrounds regardless of geographic location. Notably, even one of the studies carried out in private schools in Badagry was found to lack a fully enabling environment for menstrual hygiene management, despite their relative advantage over public schools [32], indicating that the public-private disparity, while significant, should not obscure the fact that institutional menstrual support is inadequate across school types. This public-private disparity was consistent across multiple study sites, showing that inadequate WASH was closely linked to girls’ inability to change materials, fear of staining, and school absenteeism, and that it must be addressed as an equity issue within education policy specifically [45, 46]. Furthermore, evidence on whether menstrual health interventions are associated with reduced absenteeism is mixed. A recent systematic review on mixed evidence on menstrual interventions and absenteeism found that while six of nine relevant LMIC studies reported positive intervention effects on absenteeism, three cluster-randomised controlled trials found no effect, partly because absenteeism measures were not standardised and absence due to menstruation was difficult to isolate from other causes such as illness or financial barriers to schooling [42]. This inconsistency in the evidence necessitates caution regarding the educational impact of menstrual interventions, highlighting an urgent need for more rigorous evaluations. Additionally, another study that focused specifically on menstrual education for younger adolescents shows that well-designed, interactive menstrual education yields greater gains than passive information delivery [47], reinforcing that high awareness alone is insufficient without structured, supportive education and adequate facilities.

At the structural level, our study identified economic disruption as a driver worsening menstrual product insecurity. A macro-level analysis study shows unemployment and low productive investment as key drivers of persistent poverty, suggesting that inadequate income and precarious livelihoods are fundamental upstream determinants of menstrual product insecurity [48–50]. Another cost analysis study on MHM further illustrates that lifetime menstrual hygiene expenditure remains substantial relative to typical Nigerian incomes, providing a structural explanation for the high prevalence of period poverty despite lower absolute costs than in high-income countries [52]. Further findings show that current responses in Nigeria remain fragmented, with no comprehensive national menstrual health framework, weak implementation of poverty alleviation measures, and limited integration of menstrual health into education and social protection systems [10, 51]. Addressing period poverty, therefore, demands coordinated multi-level action: structural poverty reduction; targeted product subsidies; large-scale WASH investment, particularly in schools and rural areas; interactive menstrual education; stigma-reduction advocacy; and full integration of menstrual health into public health, education, and social protection frameworks [43, 45, 47, 52].

### Strengths and Limitations

This review has several methodological strengths. First, it was conducted in accordance with established guidance for scoping reviews, including the Arksey and O’Malley framework, the JBI methodological guidance, and the PRISMA-ScR reporting guideline, ensuring methodological rigour and transparent reporting. Second, a comprehensive search strategy was implemented across multiple electronic databases using predefined eligibility criteria to maximise the identification of relevant studies. Third, four reviewers independently selected studies using the Rayyan systematic review platform, reducing the likelihood of selection bias through blinded screening and consensus-based resolution of disagreements. Collectively, these methodological approaches enhance the credibility, transparency, and reproducibility of the review findings.

Despite these strengths, several limitations should be considered when interpreting the findings. First, although the review targeted women aged 10 to 49 years, the available evidence was heavily concentrated among adolescent schoolgirls in secondary school settings, with limited representation of adult women, out-of-school adolescents, women in workplaces, women living with disabilities, internally displaced women, and other marginalized populations. The findings may therefore not fully reflect menstrual health experiences across all reproductive-aged women in Nigeria. Second, most included studies employed cross-sectional designs with self-reported data, limiting causal inference and introducing potential recall and social desirability bias. Convenience and purposive sampling in several studies further constrains the generalizability of findings. Third, the distribution of included studies was uneven across Nigeria’s geopolitical zones. The South-West had the highest representation (*n* = 6), followed by the South-South (*n* = 5) and South-East (*n* = 2), while the North-Central and North-West each contributed only a single study and the North-East was entirely unrepresented in the included literature. This geographic skew reflects a systemic evidence gap in the regions where the burden of period poverty is likely most acute, particularly given the documented influence of conservative gender norms, humanitarian displacement, and limited infrastructure across northern Nigeria. Many included studies were also conducted within specific states or local government areas, which further cautions against generalizing findings across Nigeria’s diverse sociocultural and infrastructural contexts. Fourth, important dimensions of period poverty remained insufficiently explored in the literature, including its intersection with gender-based violence, disability, mental health outcomes, policy implementation, humanitarian settings, and long-term economic consequences. Fifth, consistent with JBI guidance, no formal critical appraisal was undertaken because the primary purpose of this scoping review was evidence mapping rather than assessment of methodological quality. Nevertheless, the predominance of cross-sectional surveys, self-reported outcomes, and geographically concentrated studies should be considered when interpreting the findings. Sixth, although Google Scholar was included to improve retrieval of grey literature, its proprietary ranking algorithm limits complete reproducibility of search results. Restricting screening to the first 200 records may have resulted in some relevant publications not being identified. Seventh, restricting eligibility to studies by publication year may have excluded earlier Nigerian research that contributed to understanding menstrual hygiene management and period poverty. Consequently, the review may not fully capture historical trends in menstrual health research within the country. Eighth, although reviewer calibration exercises were conducted before screening, formal inter-reviewer agreement statistics such as Cohen’s kappa were not calculated. Ninth, although grey literature was included, publication bias cannot be completely excluded. Studies reporting significant or positive findings are generally more likely to be published than studies reporting null or inconclusive findings, potentially influencing the evidence synthesized in this review. Lastly, despite comprehensive searches of electronic databases, Google Scholar, organizational websites, and grey literature sources, some locally disseminated reports, institutional documents, conference proceedings, or unpublished studies may not have been retrieved. Therefore, the available evidence may not fully represent all menstrual health research conducted within Nigeria.

### Evidence gaps and research priorities

A key contribution of this scoping review is the identification of important gaps in the Nigerian literature on period poverty. Although interest in menstrual health has grown in recent years, the available evidence remains narrow in scope, geographically uneven, and concentrated among specific population groups. Addressing these gaps is essential for developing policies and interventions that respond to the realities of all women and girls in Nigeria.

First, the evidence base is heavily centred on adolescent schoolgirls, particularly those attending secondary schools. While this population remains important, there is limited research on adult women, out-of-school adolescents, women in workplaces, married women, and women in tertiary institutions. As a result, the menstrual experiences of a large proportion of Nigerian women of reproductive age remain poorly understood.

Second, there is a notable lack of evidence on vulnerable and marginalized populations. Very few studies have explored period poverty among women and girls living with disabilities, internally displaced persons, women in humanitarian settings, conflict-affected populations, or those experiencing extreme socioeconomic deprivation. Given the multiple barriers these groups face, their exclusion represents a significant gap in literature.

Third, important geographical disparities exist within the evidence base. Most studies were conducted in Southern Nigeria, particularly in the South-West, while evidence from Northern Nigeria remains scarce. The complete absence of studies from the North-East and the limited representation of the North-West and North-Central regions restrict understanding of how cultural norms, insecurity, displacement, and infrastructural challenges shape menstrual experiences in these settings.

A further gap relates to study design. The overwhelming majority of included studies employed cross-sectional approaches, providing only a snapshot of menstrual experiences at a single point in time. Consequently, little is known about the long-term effects of period poverty or the sustainability and effectiveness of interventions aimed at improving menstrual health outcomes. Longitudinal studies and rigorous programme evaluations remain largely absent from the Nigerian literature.

The review also revealed limited evidence on the broader consequences of period poverty beyond menstrual hygiene management. While some studies reported psychosocial challenges, there remains insufficient understanding of the relationship between period poverty and mental health outcomes such as anxiety, depression, self-esteem, emotional distress, and social exclusion. Similarly, the intersection between period poverty, gender-based violence, sexual coercion, and intimate partner vulnerability remains underexplored despite emerging evidence suggesting important links between these issues.

Another important gap concerns policy and implementation research. Although Nigeria recently adopted a National Policy on Menstrual Health and Hygiene Management, there is little empirical evidence on policy implementation, financing mechanisms, institutional accountability, or the effectiveness of existing menstrual health programmes. Understanding how policies are translated into practice will be critical for achieving sustainable improvements in menstrual health outcomes.

The economic dimensions of period poverty also remain insufficiently investigated. While affordability consistently emerged as a major barrier to menstrual product access, few studies have quantified the household economic burden of menstruation, assessed the cost-effectiveness of menstrual health interventions, or examined the broader economic consequences of period poverty for educational attainment, workforce participation, and productivity.

Future studies should investigate menstrual waste generation, disposal practices, environmentally sustainable disposal systems, and institutional waste management infrastructure across schools and communities.

Comparative implementation studies evaluating disposable and reusable menstrual products, including environmental and economic outcomes, should be prioritized.

Finally, the review identified a lack of nationally representative evidence. Most available studies were conducted within specific schools, communities, or local government areas, limiting the generalisability of findings across Nigeria’s diverse social, cultural, and economic contexts. There is a clear need for nationally representative studies and the integration of standardized menstrual health indicators into routine surveys and monitoring systems to support evidence-informed decision-making.

## Recommendations for policy

Addressing period poverty in Nigeria requires immediate action on interventions that are already supported by national policies and have the potential to produce meaningful results within a relatively short period. The first priority should be the full implementation of the National Policy on Menstrual Health and Hygiene Management, supported by clear accountability mechanisms and adequate funding. Menstrual health should also be integrated into school health programmes so that girls receive accurate information, timely support, and access to essential services throughout their education. Strengthening menstrual health education within the national curriculum will help dispel myths, reduce stigma, and promote healthy menstrual practices among both girls and boys. At the same time, improving the collection of menstrual health indicators through national surveys will provide the evidence needed to monitor progress, identify gaps, and guide policy decisions.

The second priority involves investments that build a stronger and more supportive environment for menstrual health. Schools and public institutions should be equipped with gender-responsive water, sanitation, and hygiene (WASH) facilities that provide privacy, clean water, soap, and safe disposal options for menstrual materials. Expanding subsidized menstrual product programmes, particularly for girls and women from low-income households and other vulnerable groups, would reduce financial barriers that often contribute to school absenteeism and poor menstrual hygiene. These efforts should be complemented by better teacher training and sustained community engagement to encourage open discussions, reduce harmful cultural beliefs, and create supportive environments for menstruating individuals. Encouraging local production of affordable menstrual products can also improve availability while reducing costs and creating economic opportunities.

Over the longer term, menstrual health should be embedded within broader national development strategies rather than being treated as a stand-alone issue. Integrating menstrual health into poverty reduction initiatives and social protection programmes can help address the underlying economic challenges that prevent many girls and women from managing menstruation safely and with dignity. Sustainable progress will also depend on stronger collaboration across sectors, including health, education, gender, water and sanitation, and finance. Coordinated planning, shared financing, and clearly defined responsibilities among these sectors will improve programme efficiency and ensure that menstrual health receives sustained policy attention.

Finally, continued investment in implementation research and programme evaluation is essential to identify the most effective and cost-efficient interventions in different settings. Generating high-quality local evidence will help policymakers refine existing programmes, scale up successful approaches, and ensure that resources are directed towards interventions that achieve the greatest impact. These priorities should be adapted according to available resources and local implementation contexts across Nigeria’s six geopolitical zones.

## Conclusion

This scoping review demonstrates that period poverty in Nigeria is a multidimensional challenge shaped by interacting socioeconomic, cultural, institutional, and structural determinants. The available evidence consistently links affordability, inadequate WASH infrastructure, menstrual stigma, and limited menstrual health education with poorer menstrual health experiences. However, the current evidence base is dominated by cross-sectional studies conducted among adolescent schoolgirls, highlighting important geographical and population gaps. Future research should prioritize longitudinal, intervention, and implementation studies involving underserved populations. Addressing period poverty will require coordinated action across the health, education, WASH, gender, and social protection sectors to improve menstrual health equity and dignity for women and girls in Nigeria.

## Supplementary Information

Supplementary Material 1. Search terms.

Supplementary Material 2. Extracted Data.

## Data Availability

The datasets used are provided in the supplementary materials.

## Abbreviations

COVID-19: Coronavirus Disease 2019
JBI: Joanna Briggs Institute
LMICs: Low- and Middle-Income Countries
MHM: Menstrual Hygiene Management
MeSH: Medical Subject Headings
NGO: Non-Governmental Organization
PCC: Population–Concept–Context
PRISMA-ScR: Preferred Reporting Items for Systematic Reviews and Meta-Analyses Extension for Scoping Reviews
SDG: Sustainable Development Goals
SEM: Socioecological Model
SSA: Sub-Saharan Africa
WASH: Water, Sanitation and Hygiene
