# Supplementary material for: Period poverty in Nigeria: a scoping review of its drivers and implications among women of reproductive age
Source: BMC Public Health. 2026 Aug 8;26:3202. doi: 10.1186/s12889-026-28966-x (PMC13733061; doi:10.1186/s12889-026-28966-x)
Supplement: Supplementary file 1 [file 12889_2026_28966_MOESM1_ESM.docx]

**Databases and Search Strings**

Three electronic databases were searched: Google Scholar, PubMed, and Scopus.

**Google**

("menstrual health" OR "menstrual product access" OR "menstrual education" OR "menstrual hygiene" OR" period poverty" OR "menstrual poverty" OR "period equity") AND ( "Nigeria")

**PubMed**

("menstrual health" OR "menstrual product access" OR "menstrual education" OR "menstrual hygiene" OR" period poverty" OR "menstrual poverty" OR "period equity" OR "Menstruation"[MeSH] OR "Hygiene"[MeSH] OR "Menstrual Hygiene Products"[MeSH]) AND ( "Nigeria")

**Scopus**

("menstrual health" OR "menstrual product access" OR "menstrual education" OR "menstrual hygiene" OR" period poverty" OR "menstrual poverty" OR "period equity") AND ( "Nigeria")
